# Impact of obesity on dental implant failure and peri-implant health: a systematic review and meta-analysis

**DOI:** 10.1186/s12903-026-07908-4

**Published:** 2026-02-16

**Authors:** Mingfu Ye, Zhang Wu, Xiuwen Lin, Wenjun Liu, Lihui Yan, Hom-Lay Wang

**Affiliations:** 1 Implantology Department, Xiamen Stomatological Hospital of Xiamen Medical College, Xiamen, China; 2 Shanghai Key Lab of D&A for Metal-Functional Materials, School of Materials Science & Engineering, Tongji University, Shanghai, China; 3 Department of Prosthodontics, Xiamen Stomatological Hospital of Xiamen Medical College, Xiamen, China; 4 Department of Periodontics, Xiamen Stomatological Hospital of Xiamen Medical College, Xiamen, China; 5 Department of Periodontics and Oral Medicine, University of Michigan School of Dentistry, 1011 North University Avenue, Ann Arbor, Michigan 48109-1078 USA

**Keywords:** Body mass index, Overweight, Obese, Bone loss, Probing depth

## Abstract

**Objective:**

Obesity is a growing epidemic worldwide; however, its impact on dental implant outcomes remains unclear. We systematically reviewed the evidence of the effects of body mass index (BMI) on the risk of implant failure and peri-implant health indices.

**Methods:**

PubMed, Embase, Scopus, and Web of Science literature databases were searched for studies assessing the impact of BMI on dental implant failure and peri-implant health indices up to June 10, 2025. Implant failure was the primary outcome, while plaque index, bleeding on probing (BOP), probing depth, and marginal bone loss (MBL) were secondary outcomes. The quality of the studies was assessed using the Newcastle-Ottawa Scale for cohort studies and the JBI Critical Appraisal Checklist for Cross-Sectional Studies. Odds ratios (OR) with 95% confidence intervals (CI) were calculated for dichotomous data. Mean difference (MD) or Standardised MD (SMD) were generated for continuous variables.

**Results:**

Eleven studies were included in the review, and nine were available for the meta-analysis. Meta-analysis showed that there was no statistically significant difference in the risk of implant failure between obese and non-obese groups (OR: 0.73 95% CI: 0.29, 1.86 I^2^ = 67%). However, pooled analysis showed that obese patients had significantly higher plaque index values (SMD: 2.24 95% CI: 0.84, 3.64 I^2^ = 95%), higher probing depth (MD: 1.56 95% CI: 1.32, 1.80 I^2^ = 95%), higher BOP rates, (SMD: 2.89 95% CI: 1.25, 4.53 I^2^ = 97%) and greater MBL (MD: 1.20 95% CI: 0.67, 1.72 I^2^ = 99%) as compared to non-obese patients. Sensitivity analyses confirmed the consistency of the results. Certainty of evidence was ‘very low’ for all outcomes.

**Conclusions:**

Low-quality evidence from a limited number of predominantly retrospective studies suggests that obesity may not significantly affect implant failure rates. However, it is associated with worse peri-implant health indices. Further high-quality, prospective studies are necessary to strengthen the evidence base.

**Supplementary Information:**

The online version contains supplementary material available at 10.1186/s12903-026-07908-4.

## Introduction

The World Health Organisation defines overweight and obesity as abnormal or excessive fat accumulation with a body mass index (BMI) ≥ 30 kg/m², which may impede health [1]. The prevalence of obesity has reached the point that it has become a worldwide epidemic, affecting 2.5 billion adults who are now overweight and 890 million people who are classified as obese [2]. Obesity has risen exponentially in the past two decades, and it is generally acknowledged as an independent predictor of worse health consequences [3]. It is anticipated that by 2030, approximately half of the global population will be obese [4].

Obesity is a known risk factor for several systemic conditions, like hypertension, dyslipidemia, and cardiovascular disorders like myocardial infarction and stroke. It is also linked with several malignancies, joint problems, as well as mental health disorders [5, 6]. Adipose tissue hypertrophy may lead to increased synthesis of pro-inflammatory adipokines and macrophage infiltration [7]. Ectopic fat deposition is observed in lean tissues, such as the heart, liver, pancreas, and kidneys, in obese individuals. These factors, along with increased adipose tissue mass, create a pro-inflammatory and insulin-resistant environment that leads to the development of several systemic disorders [8]. In fact, elevated levels of pro-inflammatory cytokines and periodontal pathogens in obese patients have also been connected to the development of periodontal disease [9]. A systematic review has considered waist circumference and obesity as potential risk factors for adverse periodontal disease indices [10].

Despite the substantial number of implants being used in clinical settings, it remains uncertain whether the biological mechanisms through which obesity influences periodontitis also apply to peri-implant conditions. Previously, only one systematic review by Monteiro et al. [11] has reported the association between obesity and dental implant outcomes, but with the inclusion of just six studies. In light of the growing concerns regarding the high prevalence of overweight and obesity globally, as well as the increasing number of patients seeking dental implant treatment, robust and high-quality evidence must be generated regarding the impact of obesity on implant outcomes. Hence, we conducted this updated systematic review and meta-analysis to examine the effects of obesity on implant failure and peri-implant health indices. Implant failure was the primary outcome, while plaque index, bleeding on probing (BOP), probing depth, and marginal bone loss (MBL) were secondary outcomes.

## Materials and methods

We followed the Preferred Reporting Items for Systematic Reviews and Meta-Analysis (PRISMA) statement for the review [12]. The meta-analysis protocol was registered on PROSPERO under the number CRD420251057701. This is a systematic review and meta-analysis; therefore, human ethics and consent-to-participate declarations are not applicable.

### Eligibility criteria

The PECO framework was adopted to establish the study’s inclusion criteria. We included studies conducted on a ‘*Population’* of dental implant patients. Studies were to use obesity based on BMI as the *‘Exposure’* variable and *‘Compare’* such patients with a group of non-obese individuals. Studies were to report implant failure or peri-implant health indices as *‘Outcomes’*. No restrictions were placed on study design or language of publication.

We excluded studies focusing only on obese patients without a control group, not segregating patients based on BMI, using any other obesity indicator, and published only as abstracts.

### Search and selection of studies

A comprehensive search of the PubMed, Embase, Scopus, and Web of Science literature databases was conducted by two independent reviewers (LHY and WJL). Studies that were published between the establishment of these databases and June 10, 2025, were eligible. Keywords utilised included obesity, body mass index, overweight, and dental implants. The PubMed search string was: (((obese) OR (obesity)) OR (body mass index)) OR (BMI)) OR (overweight)) AND (dental implant). Similar search queries were run in the remaining databases (Supplementary Table 1). Additional investigations were identified by meticulously reviewing the references of all articles included. A Google Scholar search was also conducted to locate papers in the gray literature.

**Fig. 1:**
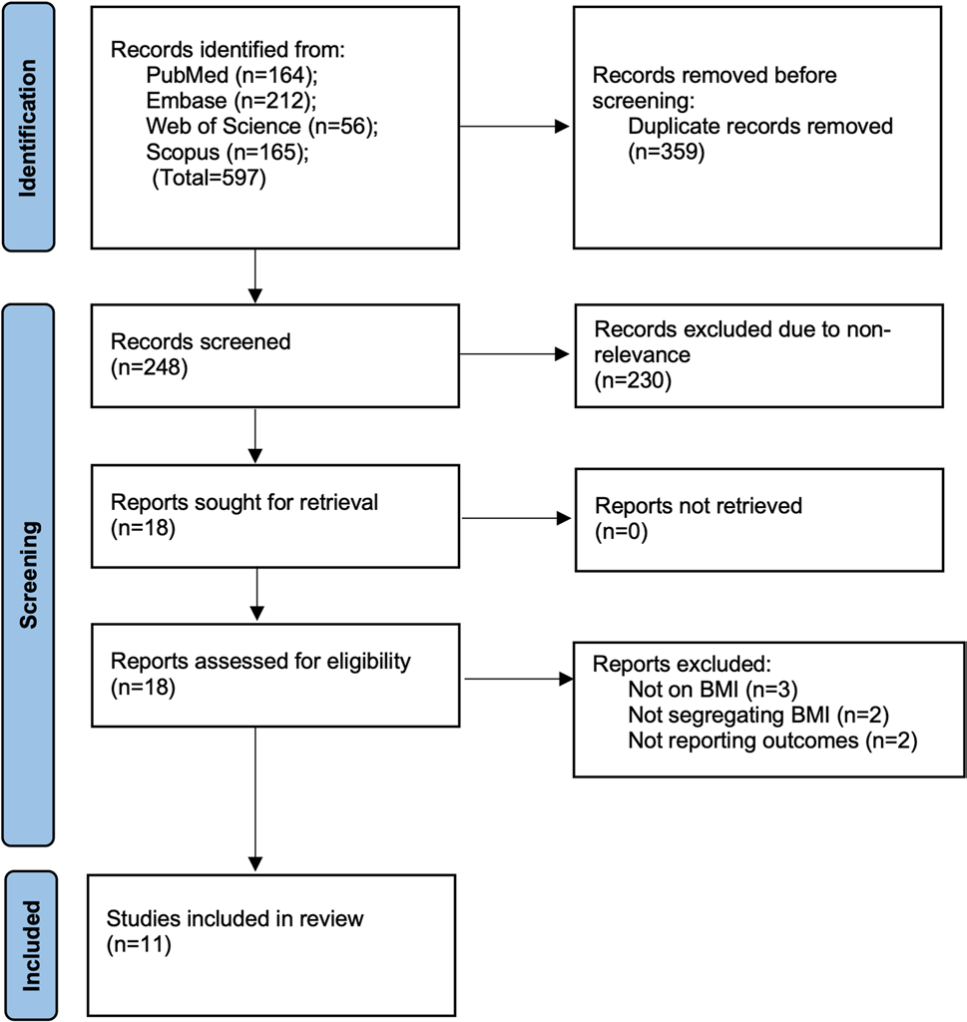
Study flowchart

After importing the database-searched articles into EndNote version X9 (Thomson Reuters, New York, NY, USA), we removed repetitive studies. Subsequently, the same two evaluators (LHY and WJL) conducted independent assessments of the studies to determine their inclusion in the review. This was achieved through a detailed examination of the abstracts and titles of the articles. Relevant studies that either reviewer identified were subsequently subjected to a thorough text analysis before inclusion (Fig. 1). The two reviewers’ disagreements were ultimately resolved through discussion with a third reviewer (ZW).

### Data extraction

Data extraction was performed independently by two reviewers (LHY and WJL) and then cross-checked by a third reviewer (ZW). Information obtained from the studies included details such as author, location, study design, inclusion criteria, sample size, age, gender, number of implants, loading protocol, BMI categories, implant system, follow-up, and outcomes assessed.

The study’s outcomes included implant failure and peri-implant health. All definitions reported by the studies were acceptable. Implant failure was the primary outcome, while plaque index, BOP, probing depth, and MBL were secondary outcomes. Any other outcomes reported by the studies were descriptively analysed.

### Risk of bias analysis

Quality assessment of included studies was done using the Newcastle Ottawa Scale (NOS) [13] for cohort studies and JBI Critical Appraisal Checklist for Analytical Cross-Sectional Studies (jbi.global/critical-appraisal-tools). For NOS, the two researchers (LHY and WJL) assigned a quality score to each article (ranging from zero to nine). The evaluation was conducted in three distinct areas: exposure or outcome identification, comparability of groups, and participant selection. The maximum possible points for each of the three categories is four, two, and three, respectively. Likewise, the same two reviewers assessed cross-sectional studies for the eight questions of the JBI Critical Appraisal Checklist, marking them as “yes”, “no”, “unclear” or “not applicable”. Risk of bias assessment was conducted independently by the two reviewers (LHY and WJL), and the third author (ZW) was consulted to resolve any discrepancies.

### Data analysis

We pooled outcome data in the DerSimonian and Laird random-effects meta-analysis model. Odds ratios (OR) with 95% confidence intervals (CI) were calculated for dichotomous data. The mean difference (MD) was calculated for continuous variables measured on the same scale. Outcomes, such as plaque index and BOP, were reported on differing scales; therefore, the standardized mean difference (SMD) was used. Statistical significance was considered for p-values less than 0.05, which was the threshold used for all analyses. Data analysis was conducted in “Review Manager” (Version 5.3) software. Heterogeneity among studies was assessed through Cochran’s Q statistic and the I^2^ index. I^2^ of over 50% and/or *P* < 0.05 indicated significant heterogeneity. Due to the limited number of studies, funnel plots were not used to assess publication bias. Subgroup analysis was conducted according to the study design. Sensitivity analysis was performed by removing one study at a time from the meta-analysis. Certainty of evidence was assessed using GRADE.

## Results

### Search results and study details

Initially, 597 articles were identified across databases including PubMed (164), Embase (212), Scopus (165), and Web of Science (56) (Fig. 1). Two hundred forty-eight original papers underwent preliminary screening after the removal of similar references. Two hundred thirty papers were discarded after the titles and abstracts were reviewed, leaving only 18 for the full-text review. The inclusion criteria were not met by seven studies, resulting in their elimination. Eleven studies [14–24] were included in the systematic review. There were no disputes among the reviewers regarding the selection of any study (Kappa = 1). No further studies were identified in the grey literature. All studies underwent thorough evaluation for the feasibility of data synthesis. Two studies did not report sufficient numerical data for the meta-analysis and were therefore assessed only descriptively. The remaining nine studies were included in the meta-analysis.

Baseline details of the studies are presented in Table 1. The inter-reviewer agreement for data extraction was high (Kappa = 0.9). Three studies were cross-sectional, one was a prospective cohort study, while all others were retrospective cohort studies. Countries of origin were the USA, Germany, Australia, India, and Saudi Arabia. Five studies enrolled only non-smokers and those without any significant systemic disease. Two studies used the Asian WHO classification to define obese as those with a BMI ≥ 27.5 kg/m^2^. All remaining studies defined obesity as a BMI of ≥ 30 kg/m². In the studies included in the meta-analysis, 6838 implants placed in obese patients were compared with 12,071 implants placed in non-obese patients. None of the studies adjusted for confounding factors. The follow-up of the studies ranged from 6 months to over 6 years. All outcomes reported by the studies, along with the results, are shown in Table 2. Quantitative data were reported at the implant level in the included studies.

**Table 1:** Details of included studies

Study	Location	Type	Included patients	BMI (kg/m^2^) categories	Patients (*n*)	Age (years)	Males (*n*)	Implants (*n*)	Loading protocol	Follow-up	Outcomes assessed
Elangovan 2014 [21]	USA	CS	Adult, non-smokers, enrolled in periodontal maintenance program with one rough-surface implant in function for 6 months	Obese: ≥30 OW: 25–29.9 Normal: 18.5–24.9 UW: <18.5	73 (all); 26 (obese)	NR	NR	73 (all)	NR	6 months (all)	Cytokine in PISF
Abduljabbar 2016 [19]	Saudi Arabia	RC	Adult, non-smokers, no major systemic disease, not using anti-inflammatory drugs/steroids, not received scaling past 3 months, with functional implant x 12 months	Obese: ≥30 Normal: 18.5–24.9	35 37	44.1 43.1	35 37	45 48	Delayed Delayed	6.8 years 6.2 years	Probing depth, BOP, MBL, Whole salivary IL-1 beta and IL-6
Hazem 2016 [17]	USA	RC	Adults with one functional implant and BMI records	Obese: ≥30 OW: 25–29.9 Normal: 18.5–24.9	58 83 79	54.5 58.4 54.3	31 53 33	89 122 110	NR	NR	Implant failure^a^ MBL (present/ absent) Complications (soft tissue complications and MBL)
Knabe 2017 [16]	Germany	PC	Adults undergoing sinus lift surgery with b-tricalcium phosphate graft and staged implant placement	Obese: ≥30 Non-obese: <30	NR	NR	NR	9 111	NR	6 months (all)	Implant failure (not defined)
Oztel 2017 [23]	Australia	RC	Patients receiving dental implants	Obese: ≥30 Non-obese: <30	177 (all)	60.2 (all)	NR	244 65	Both	36.3 months (all)	Implant failure^b^
Vohra 2017 [22]	Saudi Arabia	RC	> 30 years, non-smokers, no major systemic disease, implant in function for ≥ 36 months	Obese: ≥27.5 Non-obese: 18.5–22.9	59 25	50 52.1	NR	100 43	NR	> 3 years	Plaque index, Probing depth, BOP, MBL
Alkhudhairy 2018 [14]	Saudi Arabia	RC	> 30 years, non-smokers, no major systemic disease, bone height adequate to allow 3.5 × 10 mm implant	Obese: ≥27.5 Non-obese: 18.5–22.9	20 18	48.6 52.6	12 10	35 31	Delayed	5 years	Plaque index, Probing depth, BOP, MBL
Elsadek 2022 [15]	Saudi Arabia	CS	Non-smokers, no major systemic disease, not using anti-inflammatory drugs, antibiotics, not undergone periodontal procedure, implant in function for ≥ 36 months	Obese: ≥30 Non-obese: <30	40 40	43.4 41.4	30 25	38 39	Delayed	-	Plaque index, Probing depth, BOP, MBL
Mehta 2023 [18]	India	RC	Dental implants placed for missing mandibular 1st molar	Obese: ≥30 Normal: <25	40 20	51 48.3	NR	40 20	NR	NR	Probing depth, MBL
Kayal 2024 [25]	India	CS	Atleast one implant in function for > 1 year post crown, free of systemic disease, not under long-term medication, no periodontal disease or therapy	Obese: ≥30 Normal: <25	431 428	NR	NR	431 428	NR	1 year	Probing depth, MBL, CAL
Steeds 2025 [20]	USA	RC	All patients with dental implants	Obese: ≥30 OW: 25–29.9 Normal: 18.5–24.9	2161 1982 2098	NR	NR	5847 5371 5703	NR	NR	Implant failure^a^

*CS* Cross-sectional, *PC* Prospective cohort, *RC* Retrospective cohort, *UW* Underweight, *OW* Overweight, *n* number, *BOP* Bleeding on probing, *MBL* Marginal bone loss, *IL* Interleukin, *PISF* Peri-implant sulcular fluid, *NR* Not reported, *CAL* Clinical attachment loss

^a^Defined as implant removed from the patients mouth

^b^Defined as implant requiring replacement

**Table 2:** Outcomes of the included studies

Study	Outcomes	Results
Elangovan 2014 [21]	Cytokine in PISF	No statistical significant association between BMI and any cytokine level in PISF
Abduljabbar 2016 [19]	Probing depth BOP MBL Whole salivary IL-1 beta and IL-6	Significantly higher probing depth in obese individuals Significantly higher BOP in obese individuals Significantly higher MBL in obese individuals Significantly higher salivary IL-1 beta and IL-6 levels in obese individuals
Hazem 2016 [17]	Implant failure MBL Complications (soft tissue complications and MBL)	No significant association between BMI and implant failure Incidence did not significantly differ between BMI categories Obese patients had significantly higher odds to complications as compared to normal BMI
Knabe 2017 [16]	Implant failure	No significant association between BMI and implant failure
Oztel 2017 [23]	Implant failure	Significantly higher implant failure in non-obese group
Vohra 2017 [22]	Plaque index Probing depth BOP MBL	Significantly higher plaque index in obese individuals Significantly higher probing depth in obese individuals Significantly higher BOP in obese individuals Significantly higher MBL in obese individuals
Alkhudhairy 2018 [14]	Plaque index Probing depth BOP MBL	Significantly higher plaque index in obese individuals Significantly higher probing depth in obese individuals Significantly higher BOP in obese individuals Significantly higher MBL in obese individuals
Elsadek 2022 [15]	Plaque index Probing depth BOP MBL	Significantly higher plaque index in obese individuals Significantly higher probing depth in obese individuals Significantly higher BOP in obese individuals Significantly higher MBL in obese individuals
Mehta 2023 [18]	Probing depth MBL	Significantly higher probing depth in obese individuals Significantly higher MBL in obese individuals
Kayal 2024 [25]	Probing depth MBL CAL	Significantly higher probing depth in obese individuals Significantly higher MBL in obese individual Significantly higher CAL in obese individuals
Steeds 2025 [20]	Implant failure	No significant association between BMI and implant failure

*BOP* Bleeding on probing, *MBL* Marginal bone loss, *IL* Interleukin, *PISF* Peri-implant sulcular fluid, *CAL* Clinical attachment loss

### Implant failure

**Fig. 2:**
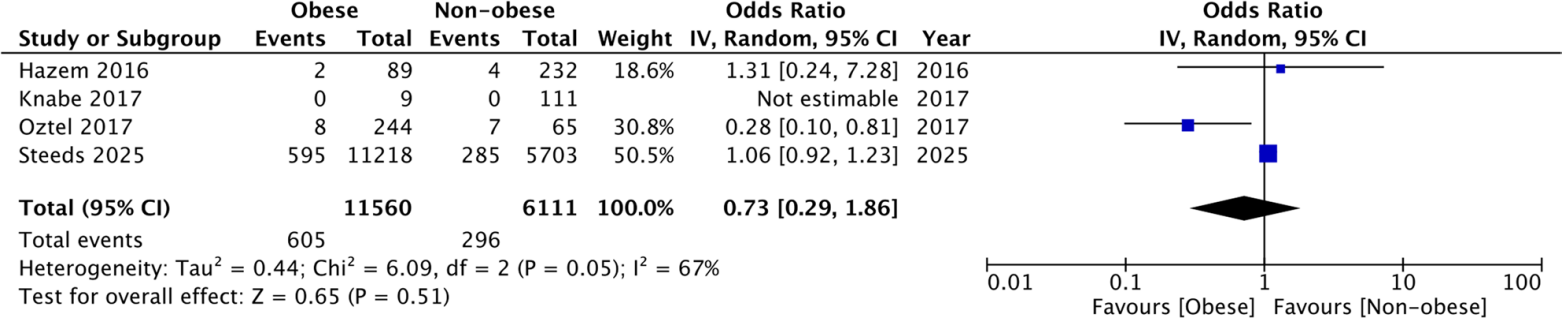
Meta-analysis of implant failure between obese and non-obese groups. IV, inverse variance; CI, confidence intervals

Four studies reported data on implant failure. Only the study of Oztel et al. [23] reported lower failure rates in the obese group as compared to the non-obese group. All the remaining studies did not find any significant association between BMI and implant failure. Meta-analysis also showed that there was no statistically significant difference in the risk of implant failure between obese and non-obese groups (OR: 0.73 95% CI: 0.29, 1.86 I^2^ = 67%) (Fig. 2). The results were stable on sensitivity analysis. Subgroup analysis is shown in Supplementary Table 2. The single prospective cohort study did not report any implant failure, and hence, the OR was not estimable.

### Peri-implant health

**Fig. 3:**
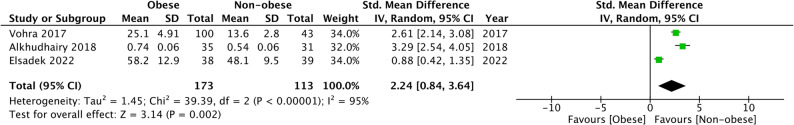
Meta-analysis of plaque index between obese and non-obese groups. IV, inverse variance; CI, confidence intervals

**Fig. 4:**
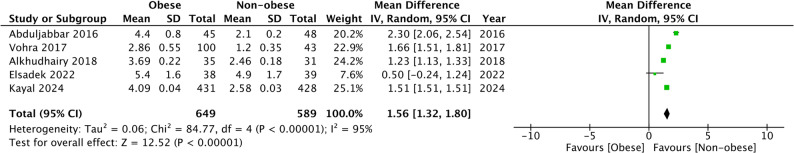
Meta-analysis of probing depth between obese and non-obese groups. IV, inverse variance; CI, confidence intervals

**Fig. 5:**
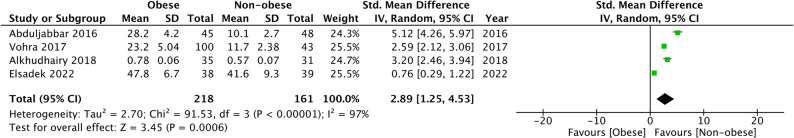
Meta-analysis of BOP between obese and non-obese groups. IV, inverse variance; CI, confidence intervals

**Fig. 6:**
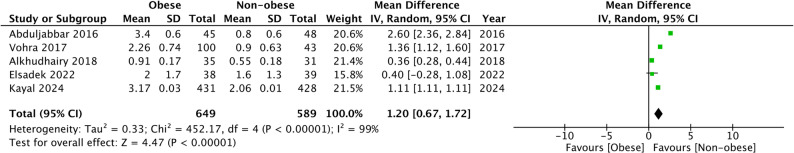
Meta-analysis of MBL between obese and non-obese groups. IV, inverse variance; CI, confidence intervals

Three studies reported data on plaque index. Meta-analysis showed that obese patients had significantly higher plaque index values as compared to non-obese patients (SMD: 2.24 95% CI: 0.84, 3.64 I^2^ = 95%) (Fig. 3). The significance of outcomes did not change when individual studies were excluded. Probing depth was reported by five studies. Pooled analysis showed that obese patients had significantly higher probing depths compared to non-obese patients (MD: 1.56, 95% CI: 1.32, 1.80, I^2^ = 95%) (Fig. 4). Results remained stable on the sensitivity analysis. Four studies reported BOP. Quantitative analysis revealed significantly higher BOP rates in obese patients compared to non-obese patients (SMD: 2.89, 95% CI: 1.25, 4.53, I^2^ = 97%) (Fig. 5). Results remained statistically significant on sequential exclusion of studies. Five studies measured MBL. A pooled analysis of the same demonstrated that MBL was significantly higher in obese patients compared to non-obese patients (MD: 1.20, 95% CI: 0.67, 1.72, I^2^ = 99%) (Fig. 6). No change in the significance of results occurred on the sensitivity analysis. Subgroup analysis based on study design revealed statistical significant results for both cross-sectional and retrospective cohort studies for all peri-implant health outcomes (Supplementary Tables 2 & Supplementary Figs. 1–5).

### Risk of bias analysis

Table 3 shows the NOS scores of cohort studies. The baseline characteristics of the obese and non-obese groups were not matched by any study, thereby introducing confounding bias. Since only crude data analysis was conducted, we scored all studies zero for comparability of groups. Hence, the NOS score of the studies was either 6 or 7, indicating moderate to poor quality.

**Table 3:** Risk of bias analysis

Study	Selection of cohort	Comparability of groups	Outcomes assessment	Total NOS score
Elangovan 2014 [21]	4	-	2	6
Abduljabbar 2016 [19]	4	-	3	7
Hazem 2016 [17]	4	-	2	6
Knabe 2017 [16]	4	-	2	6
Oztel 2017 [23]	4	-	3	7
Vohra 2017 [22]	4	-	3	7
Alkhudhairy 2018 [14]	4	-	3	7
Elsadek 2022 [15]	4	-	2	6
Mehta 2023 [18]	4	-	2	6
Kayal 2024 [25]	4	-	3	7
Steeds 2025 [20]	4	-	2	6

*NOS* Newcastle Ottawa scale

Supplementary Table 3 presents the risk-of-bias assessment for cross-sectional studies. All of the included cross-sectional studies failed to account for confounding factors. The inter-reviewer agreement for risk of bias analysis was high (kappa = 0.95).

### Certainty of evidence

Table 4 presents the certainty of the evidence, as assessed by GRADE, for all outcomes. Based on the author’s judgment, the certainty of evidence was ‘very low’ for all outcomes.

**Table 4:** GRADE assessment of evidence

	Implant failure	Plaque index	Probing depth	Bleeding on Probing	Marginal bone loss
Number of studies	4	3	5	4	5
Downgrade quality of evidence					
Risk of bias	Very serious^a^	Very serious^a^	Very serious^a^	Very serious^a^	Very serious^a^
Inconsistency	No	No	No	No	No
Indirectness	No	No	No	No	No
Imprecision	Yes^b^	No	No	No	No
Publication bias	No	No	No	No	No
Upgrade quality of evidence					
Large effect	No	No	No	No	No
Plausible confounding	No	No	No	No	No
Dose-response	No	No	No	No	No
Overall certainty of Evidence	Very low	Very low	Very low	Very low	Very low

^a^No matching of baseline characteristics between the study groups in any study

^b^Wide confidence intervals

## Discussion

This review compiles evidence from 11 studies to demonstrate the impact of obesity, as measured by BMI, on dental implant outcomes. It was noted that dental implants placed in obese patients were not associated with worse survival as compared to non-obese patients. However, dental implants in obese patients demonstrated poor peri-implant health as compared to non-obese patients. In particular, obese patients seem to have higher plaque index, probing depth, BOP, and MBL. All of these results were robust in sensitivity analysis and subgroup analysis. Notably, the certainty of evidence was ‘very low’ for all outcomes, and it was derived primarily from retrospective and cross-sectional studies. The present results align with the previous review by Monteiro et al. [11], which pooled data from six studies involving 746 patients and 986 implants to demonstrate that obesity was not associated with a significantly increased risk of implant failure but was linked to worse peri-implant health indices. Our review included five additional studies and compared the data of 6838 implants placed in obese patients with 12,071 implants placed in non-obese patients. The analysis’s statistical power was considerably enhanced by the significantly increased sample size, resulting in the most robust results to date. Moreover, these findings are in line with recent evidence that systemic metabolic conditions, such as diabetes, can impair implant stability through inflammatory and immunometabolic mechanisms [26]. This suggests that both obesity and diabetes may share common pathogenic pathways affecting peri-implant health.

Nevertheless, a notable limitation of the present meta-analysis is the substantial inter-study heterogeneity observed across all peri-implant outcome measures. The heterogeneity likely stems from variations in study design, sample characteristics, implant systems, loading protocols, follow-up durations, and methods used to assess peri-implant indices. Given the limited number of studies in the analysis and the scarcity of data, we were unable to determine the factors contributing to the high heterogeneity. A subgroup analysis could only be conducted based on the study design, which also included very few studies in each subgroup. Additionally, variations in BMI classification standards, particularly between Western and Asian populations, may have contributed to the observed heterogeneity across studies, affecting the overall pooled estimates. Most Western studies defined obesity as a BMI of ≥ 30 kg/m², while some Asian studies used the lower WHO Asian threshold of ≥ 27.5 kg/m². These differing definitions could have led to misclassification, with some individuals labelled as ‘obese’ in one population being considered simply ‘overweight’ in another. We also observed a lack of standardised reporting for peri-implant parameters like BOP and plaque index, which further complicated data synthesis. Such inconsistencies in BMI cut-offs and peri-implant parameters can introduce measurement bias and make direct comparisons difficult, potentially inflating heterogeneity and reducing the precision of pooled estimates. Additionally, differences in examiner calibration and the absence of uniform diagnostic thresholds may result in under- or overestimation of peri-implant inflammation. Clinical heterogeneity was also evident in the inclusion criteria: some studies excluded smokers or patients with systemic diseases, while others did not control for these confounders. This variability, along with the predominance of retrospective and cross-sectional study designs, limits the comparability and interpretability of the pooled outcomes.

When interpreting these findings, it is essential to consider potential confounding factors, as all combined estimates are based on crude, unadjusted data. None of the studies adjusted for baseline differences between obese and non-obese patients, and none reported effect sizes that account for key factors. This limits the ability to distinguish the true impact of obesity from other risk factors. Variables such as smoking, diabetes, oral hygiene, periodontal health, implant site, and prosthetic or loading protocols are known to affect peri-implant health and may independently influence implant success and inflammation [24, 26]. Although some studies tried to reduce confounding by excluding smokers or medically compromised patients, residual confounding remains unavoidable in observational research. As a result, the associations observed, such as the notably poorer peri-implant indices in obese patients, may partly stem from these uncontrolled factors rather than a direct effect of obesity itself.

The associations observed in this review, relating obesity to poorer peri-implant health, can be better understood through the lens of the chronic low-grade systemic inflammation that characterises obesity [27]. Excess fat tissue acts as an active endocrine organ, releasing higher levels of pro-inflammatory cytokines such as IL-1β, IL-6, and TNF-α, which circulate throughout the body and can affect local tissue responses [28]. Evidence from human [21] and animal studies [29] shows that obesity creates a pro-inflammatory immunometabolic state that hampers bone healing, disturbs the balance between osteoblasts and osteoclasts, and increases the risk of peri-implant soft tissue inflammation. Supporting this, two of the included studies [14, 15] found significantly higher levels of inflammatory biomarkers in the peri-implant sulcular fluid and saliva of obese individuals, indicating a heightened inflammatory burden. The consistently elevated plaque index, probing depth, BOP, and marginal bone loss in obese patients observed in this meta-analysis likely reflect local signs of this systemic inflammation. Thus, the biological plausibility that obesity-related systemic inflammation impairs peri-implant health reinforces the mechanistic understanding of our findings and highlights the importance of considering metabolic inflammation as a key factor in maintaining implant health.

The influence of obesity on peri-implant health might also be viewed as an extension of its effect on periodontal tissues. The subgingival biofilm of both periodontitis and healthy patients has shown higher proportions of periodontal pathogens in obese individuals [30, 31]. Moreover, elevated BMI has been associated with increased concentrations of inflammatory mediators in the gingival crevicular fluid and serum of individuals with periodontitis and periodontal health. In contrast, periodontitis itself appears to influence the circulating levels of specific adipose tissue-derived mediators [32, 33]. A meta-analysis by Kim et al. [34] involving 37 studies has shown that obesity increases the odds of periodontitis by 35% regardless of age and country of origin. A recent 10-year retrospective analysis also suggests that obesity is a significant risk factor for periodontitis progression; however, the association failed to maintain statistical significance when other confounders were taken into account [9]. Indeed, the impact of confounding factors in our review cannot be underestimated. Diabetes, smoking, and alcohol consumption are major risk factors for peri-implantitis [35]. All of the studies scored low in quality, primarily because baseline characteristics were not matched, or adjusted data were not presented, especially for peri-implant indices.

Our current review exclusively considers BMI as a measure of obesity. Even though it is a commonly used tool, it may not be a reliable indicator of obesity. The weight of muscular mass in the upper limbs, lower limbs, and chest may lead to misclassification, especially in males [36]. As an alternative to BMI, waist circumference is widely recognized and routinely employed as a reliable metric for assessing central obesity. This measurement directly indicates central obesity or visceral fat accumulation [37]. Research suggests that waist circumference is a better predictor of visceral adipose tissue than BMI [38]. Recently, further metrics, including waist-to-hip and waist-to-height ratios, have been developed and are intended to provide a better prediction of central obesity than waist circumference alone [39]. However, since the majority of studies in the literature have used BMI as a surrogate marker for obesity, we had to restrict the review to BMI. We were unable to include waist circumference as a measure. Only further studies using these parameters to assess obesity in dental implant patients can provide robust evidence on the actual effects of central obesity on implant failure and peri-implant health.

The following limitations should be considered when evaluating the current evidence. Despite being an updated systematic review and meta-analysis, we could include only 11 studies in the descriptive analysis and even fewer in the meta-analysis. The number of studies was insufficient for a robust analysis of the impact of BMI on dental implant outcomes. Moreover, the absence of RCTs and the predominance of retrospective and cross-sectional data are significant drawbacks of our review. We could compare only obese with non-obese individuals and failed to conduct a subgroup analysis for overweight patients due to a lack of data. BMI categories were not uniform across studies. Among them, some Asian studies use the WHO Asian classification, while others use the Western classification to define obesity. The high inter-study heterogeneity across the meta-analyses cannot be ignored. Given the substantial heterogeneity observed, the pooled results should be interpreted with caution. We were unable to assess publication bias in our review due to the limited number of studies. However, it is plausible that studies reporting significant results may have been more likely to be published over those reporting non-significant results. It is noteworthy that the follow-up duration varied across studies, and we were unable to differentiate between early and late implant failures. It is plausible that implant failure events were underreported in studies with shorter follow-up periods. Moreover, most of the data on peri-implant indices came from a single country, and therefore, the results cannot be generalised. Lastly, GRADE assessment of evidence indicated that the certainty of evidence was “very low” for all outcomes. Very low certainty means that the current findings provide only limited confidence in the direction or magnitude of the association between obesity and peri-implant outcomes. Consequently, clinicians should avoid making firm clinical decisions based solely on these results, and researchers should prioritise well-designed prospective studies with proper confounder control to generate more reliable and generalisable evidence.

The current findings have important clinical implications. We believe that physicians should consider obesity as a modifying factor during treatment planning of dental implants. Individualised maintenance regimens, such as more frequent follow-up appointments, emphasising oral hygiene guidelines, and careful observation for early signs of peri-implant inflammation, may be beneficial for obese patients. Considering the heightened peri-implant probing depth, BOP, and MBL noted in obese persons, it is imperative to prioritize the management of systemic inflammation through interdisciplinary cooperation with medical professionals. Future research should be conducted in the form of randomised controlled trials and prospective cohort study designs that adequately account for potential confounding factors, such as smoking, diabetes, and dental hygiene habits. Furthermore, including alternative and more accurate anthropometric metrics—such as waist circumference, waist-to-hip ratio, or body composition analysis—could yield enhanced understanding of the correlation between central obesity and peri-implant outcomes. Standardisation of outcome definitions, extended follow-up periods, and multicenter trials involving diverse populations are necessary to enhance the reliability and generalizability of the existing evidence base.

## Conclusions

Low-quality evidence from a small number of mostly retrospective studies suggests that obesity may not increase implant failure risk but is associated with worse peri-implant health, thereby requiring careful monitoring.

## Supplementary Information

Supplementary Material 1. Supplementary Table 1: Search strategy.

Supplementary Material 2. Supplementary Table 2: Subgroup analysis based on study design.

Supplementary Material 3. Supplementary Table 3: Risk of bias assessment of cross-sectional studies using the JBI critical appraisal checklist.

Supplementary Material 4. Supplementary Fig. 1: Subgroup analysis of implant failure between obese and non-obese groups. IV, inverse variance; CI, confidence intervals.

Supplementary Material 5. Supplementary Fig. 2: Subgroup analysis of plaque index between obese and non-obese groups. IV, inverse variance; CI, confidence intervals.

Supplementary Material 6. Supplementary Fig. 3: Subgroup analysis of probing depth between obese and non-obese groups. IV, inverse variance; CI, confidence intervals.

Supplementary Material 7. Supplementary Fig. 4: Subgroup analysis of BOP between obese and non-obese groups. IV, inverse variance; CI, confidence intervals.

Supplementary Material 8. Supplementary Fig. 5: Subgroup analysis of MBL between obese and non-obese groups. IV, inverse variance; CI, confidence intervals.

## Data Availability

The data that support the findings of this study are available from the corresponding author upon reasonable request.

## Abbreviations

BMI: Body mass index
BOP: Bleeding on probing
CI: Confidence interval
GRADE: Grading of Recommendations, Assessment, Development and Evaluation
MBL: Marginal bone loss
MD: Mean difference
NOS: Newcastle–Ottawa Scale
OR: Odds ratio
PRISMA: Preferred Reporting Items for Systematic Reviews and Meta-Analyses
SMD: Standardised mean difference
