# Supplementary material for: Impact of obesity on dental implant failure and peri-implant health: a systematic review and meta-analysis
Source: BMC Oral Health. 2026 Feb 16;26:515. doi: 10.1186/s12903-026-07908-4 (PMC13011362; doi:10.1186/s12903-026-07908-4)
Supplement: Supplementary file 1 — Supplementary Material 1. Supplementary Table 1: Search strategy. [file 12903_2026_7908_MOESM1_ESM.docx]

Supplementary Table 1: Search strategy

- PubMed

(((((obese) OR (obesity)) OR (body mass index)) OR (BMI)) OR (overweight)) AND (dental implant)

- Embase

1. ‘obese’ OR ‘obesity’ OR ‘BMI’ OR ‘body mass index’ OR ‘overweight’

2. ‘dental implants’

3. #1 AND #2

- Scopus

(TITLE-ABS-KEY-AUTH ((obese) OR (Obesity) OR (BMI) OR (Body mass index) OR (Overweight)) AND (TITLE-ABS-KEY-AUTH(Dental implant))

- Web of Science

(((((obese) OR (obesity)) OR (body mass index)) OR (BMI)) OR (overweight)) AND (dental implant)
