# Supplementary material for: Impact of obesity on dental implant failure and peri-implant health: a systematic review and meta-analysis
Source: BMC Oral Health. 2026 Feb 16;26:515. doi: 10.1186/s12903-026-07908-4 (PMC13011362; doi:10.1186/s12903-026-07908-4)
Supplement: Supplementary file 2 — Supplementary Material 2. Supplementary Table 2: Subgroup analysis based on study design. [file 12903_2026_7908_MOESM2_ESM.docx]

Supplementary Table 2: Subgroup analysis based on study design

| Outcome | Groups | Studies | Effect size | I^2^ (%) |
| --- | --- | --- | --- | --- |
| Implant failure | Prospective cohort  Retrospective cohort | 1  3 | Not estimable  OR: 0.73 [0.29, 1.86] | -  67 |
| Plaque index | Cross-sectional  Retrospective cohort | 1  2 | SMD: 0.88 [0.42, 1.35]  SMD: 2.88 [2.22, 3.55] | -  56 |
| Probing depth | Cross-sectional  Retrospective cohort | 2  3 | MD: 1.08 [0.09, 2.06]  MD: 1.72 [1.18, 2.26] | 86  97 |
| Bleeding on Probing | Cross-sectional  Retrospective cohort | 1  3 | SMD: 0.76 [0.29, 1.22]  SMD: 3.60 [2.19, 5.01] | -  92 |
| Marginal bone loss | Cross-sectional  Retrospective cohort | 2  3 | MD: 0.84 [0.16, 1.52]  MD: 1.44 [0.08, 2.79] | 76  99 |

OR, odds ratio; SMD, standardized mean difference; MD, mean difference
