# Supplementary material for: Impact of obesity on dental implant failure and peri-implant health: a systematic review and meta-analysis
Source: BMC Oral Health. 2026 Feb 16;26:515. doi: 10.1186/s12903-026-07908-4 (PMC13011362; doi:10.1186/s12903-026-07908-4)
Supplement: Supplementary file 3 — Supplementary Material 3. Supplementary Table 3: Risk of bias assessment of cross-sectional studies using the JBI critical appraisal checklist. [file 12903_2026_7908_MOESM3_ESM.docx]

Supplementary Table 3: Risk of bias assessment of cross-sectional studies using the JBI critical appraisal checklist

|  | Elangovan 2014 | Elsadek 2022 | Kayal 2024 |
| --- | --- | --- | --- |
| Were the criteria for inclusion in the sample clearly defined? | Yes | Yes | Yes |
| Were the study subjects and the setting described in detail? | Yes | Yes | Yes |
| Was the exposure measured in a valid and reliable way? | Yes | Yes | Yes |
| Were objective, standard criteria used for  measurement of the condition? | Yes | Yes | Yes |
| Were confounding factors identified? | No | No | No |
| Were strategies to deal with confounding factors  stated? | Not applicable | Not applicable | Not applicable |
| Were the outcomes measured in a valid and reliable way? | Yes | Yes | Yes |
| Was appropriate statistical analysis used? | Yes | Yes | Yes |
