# Supplementary figures and images for: Impact of obesity on dental implant failure and peri-implant health: a systematic review and meta-analysis
Source: BMC Oral Health. 2026 Feb 16;26:515. doi: 10.1186/s12903-026-07908-4 (PMC13011362; doi:10.1186/s12903-026-07908-4)

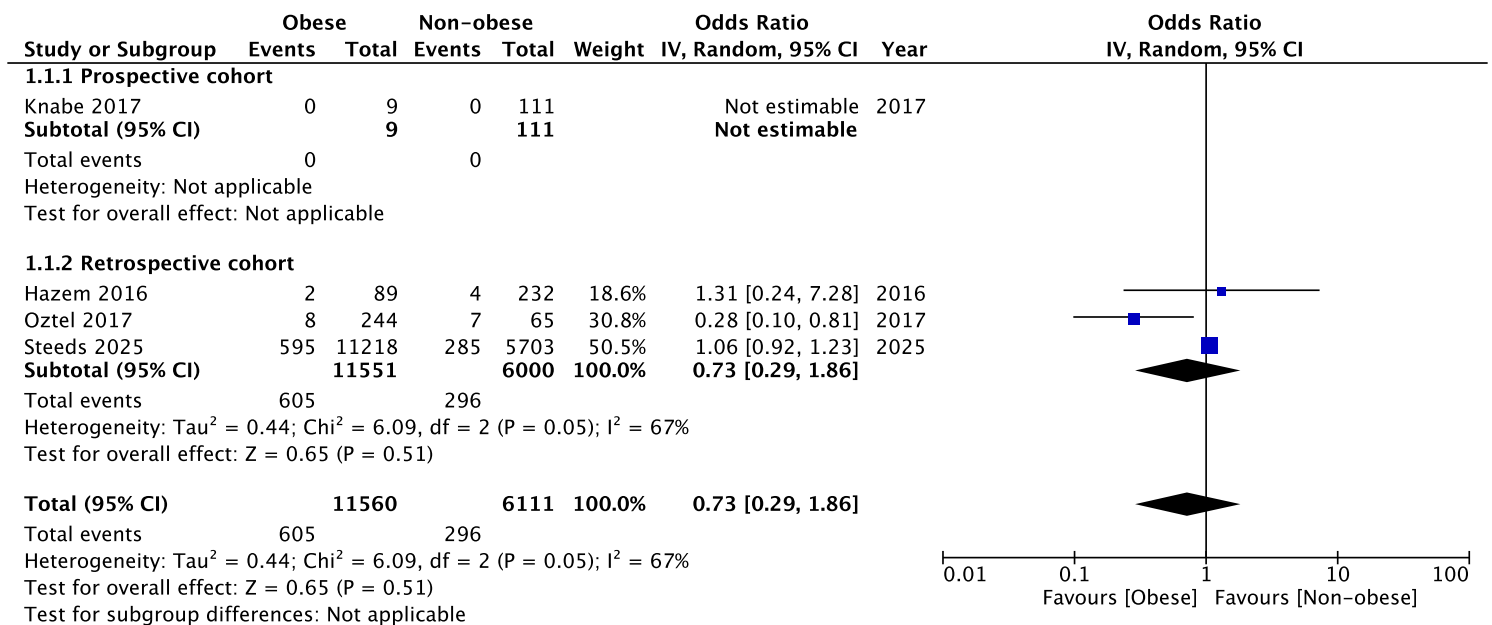

Supplement: Supplementary file 4 — Supplementary Material 4. Supplementary Fig. 1: Subgroup analysis of implant failure between obese and non-obese groups. IV, inverse variance; CI, confidence intervals. [file 12903_2026_7908_MOESM4_ESM.pdf]

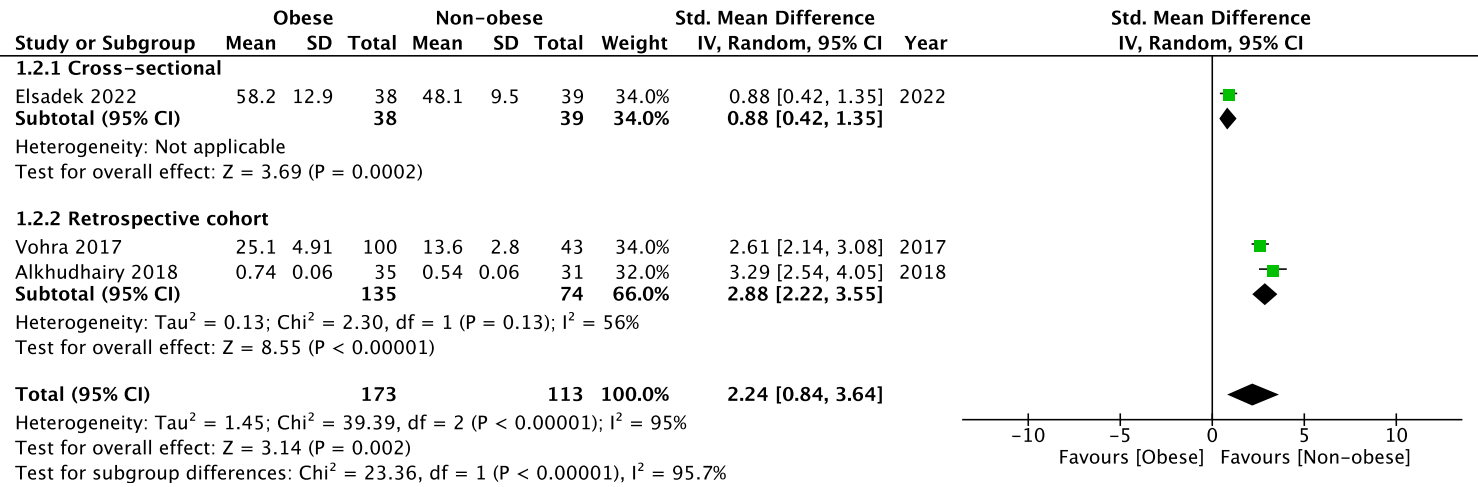

Supplement: Supplementary file 5 — Supplementary Material 5. Supplementary Fig. 2: Subgroup analysis of plaque index between obese and non-obese groups. IV, inverse variance; CI, confidence intervals. [file 12903_2026_7908_MOESM5_ESM.pdf]

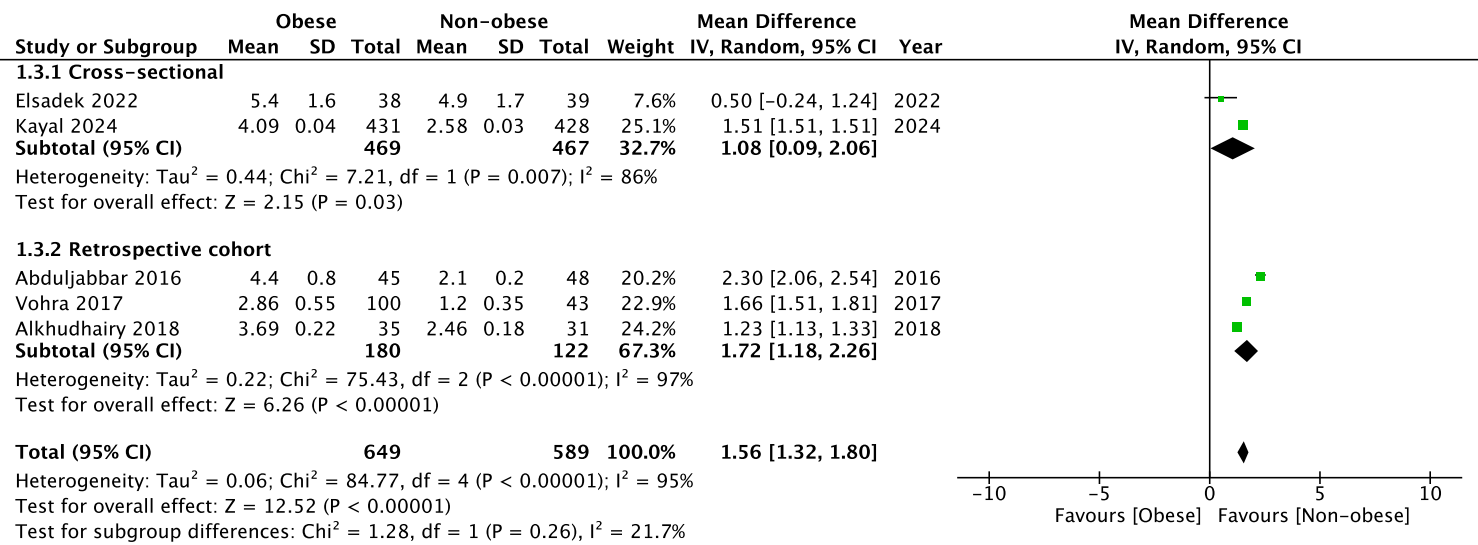

Supplement: Supplementary file 6 — Supplementary Material 6. Supplementary Fig. 3: Subgroup analysis of probing depth between obese and non-obese groups. IV, inverse variance; CI, confidence intervals. [file 12903_2026_7908_MOESM6_ESM.pdf]

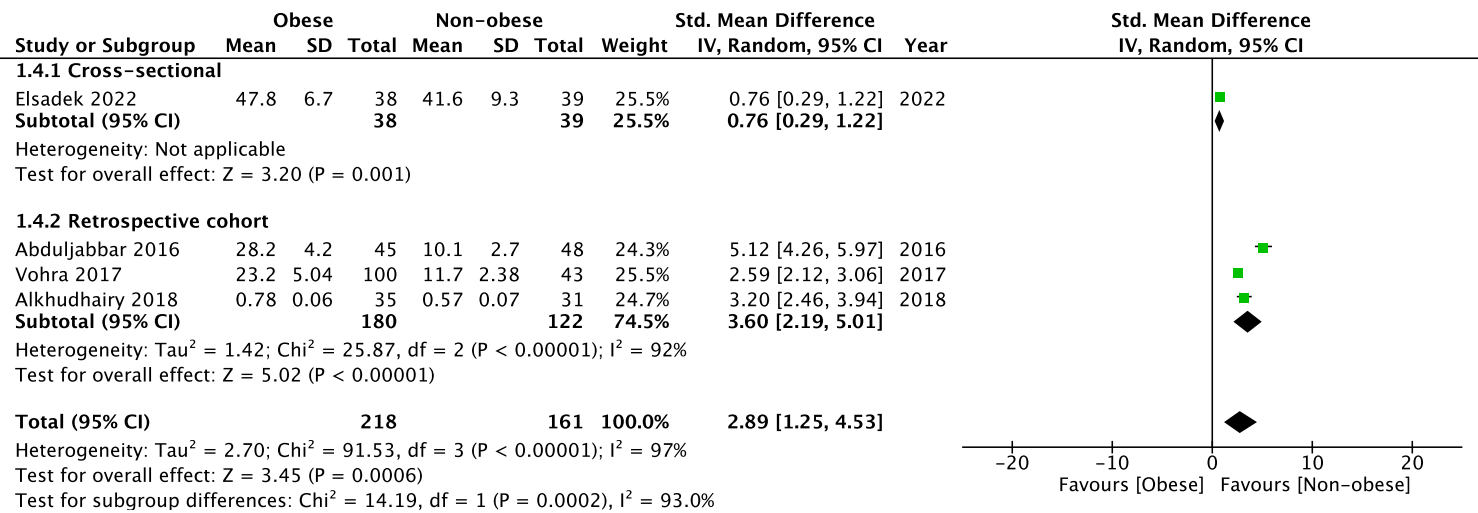

Supplement: Supplementary file 7 — Supplementary Material 7. Supplementary Fig. 4: Subgroup analysis of BOP between obese and non-obese groups. IV, inverse variance; CI, confidence intervals. [file 12903_2026_7908_MOESM7_ESM.pdf]

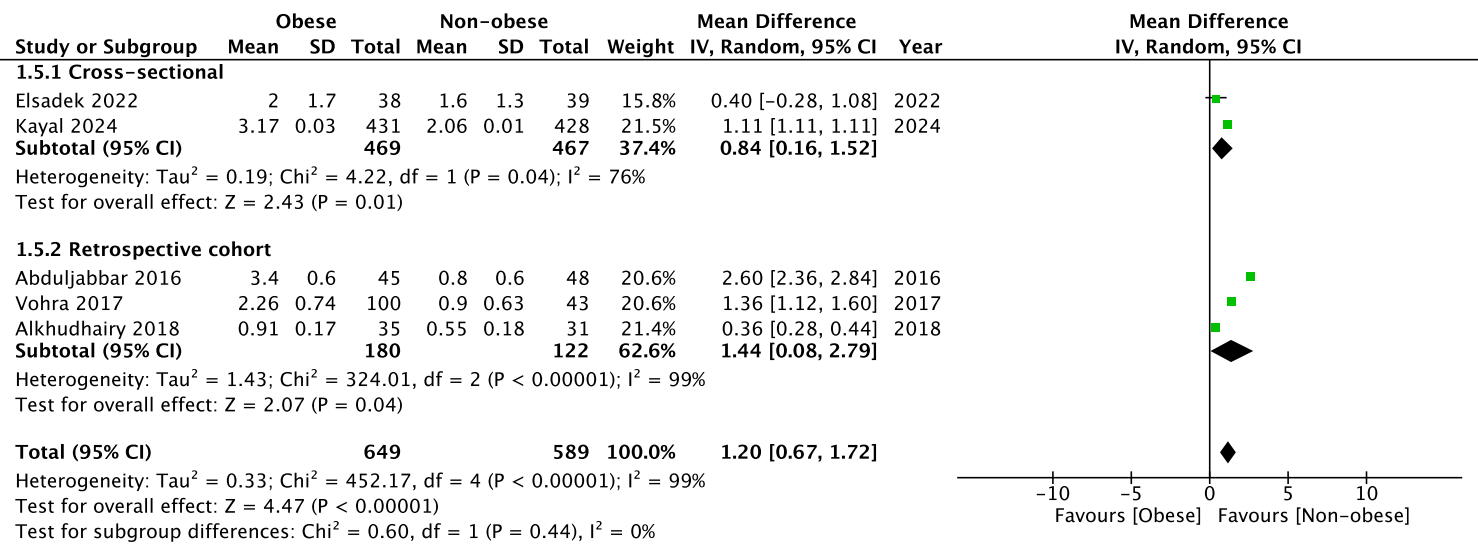

Supplement: Supplementary file 8 — Supplementary Material 8. Supplementary Fig. 5: Subgroup analysis of MBL between obese and non-obese groups. IV, inverse variance; CI, confidence intervals. [file 12903_2026_7908_MOESM8_ESM.pdf]
